# A Multi-Channel AM-TMAS Driving System Based on Amplitude-Modulated Sine Waves

**DOI:** 10.3390/bioengineering13040405

**Published:** 2026-03-31

**Authors:** Yiheng Shi, Ze Li, Ruixu Liu, Xiyang Zhang, Mingpeng Wang, Ren Ma, Tao Yin, Xiaoqing Zhou, Zhipeng Liu

**Affiliations:** 1 State Key Laboratory of Advanced Medical Materials and Devices, Tianjin 300192, China; s2023015010@pumc.edu.cn (Y.S.);; 2 Tianjin Key Laboratory of Neuromodulation and Neurorepair, Tianjin 300192, China; 3 Institute of Biomedical Engineering, Peking Union Medical College, Chinese Academy of Medical Sciences, Beijing 100730, China; 4 Institute of Biomedical Engineering, Tianjin Institutes of Health Science, Tianjin 300192, China

**Keywords:** transcranial magneto-acoustic stimulation (TMAS), amplitude modulation, rhythmic neural stimulation, deep brain stimulation, multi-channel synchronization, field-programmable gate array (FPGA)

## Abstract

Selectively modulating specific brain-rhythm bands with physical stimuli helps both to reveal neural mechanisms and to provide non-pharmacological treatment avenues for brain disorders. This study proposes and implements a multi-channel transcranial magneto-acoustic stimulation driving system based on amplitude-modulated (AM) sine waves (AM-TMAS) intended to supply a reliable hardware platform for noninvasive, focal low-frequency rhythmic electrical stimulation of deep-brain structures. The driving system implements a 64-channel AM module based on an FPGA plus high-speed DACs. Multi-channel precision is achieved via a unified high-speed clock and a global UPDATE trigger. To overcome the large separation between envelope and carrier frequencies, we developed a high-fidelity AM waveform generation method based on DDS + LUT + envelope multiplication. The algorithm first centers the carrier samples to preserve waveform symmetry, then applies LUT-based envelope coefficients and fixed-point envelope multiplication, enabling high-precision AM outputs with carrier frequencies from 100 kHz to 2 MHz and envelope frequencies from 0.1 Hz to 100 kHz. We tested the system’s rhythmic multi-channel AM output performance across frequencies and also measured magneto-acoustic-coupled rhythmic electrical signals produced by the AM-TMAS driving setup. Any single channel reliably produced high-fidelity AM waveforms with a 500 kHz carrier and 8 Hz/40 Hz envelopes; the measured carrier was 499.998 kHz with excellent frequency stability. Both envelope and carrier frequencies are flexibly tunable. At the nominal 500 kHz carrier, envelope fidelity was further quantified: the extracted envelopes achieved NRMSEs of 1.0795% (8 Hz) and 1.9212% (40 Hz), confirming high-fidelity AM synthesis. Under a 0.3 T static magnetic field, the AM-TMAS driving system generated rhythmic electrical responses in physiological saline that carried the expected 40 Hz envelope. The proposed AM-TMAS driver achieves high accuracy in AM waveform generation and robust multi-channel performance, and—when combined with an external static magnetic field—can produce rhythmically modulated magneto-acoustic electrical stimulation. This platform provides a practical technical tool for brain-function research and the development of rhythm-targeted neuromodulation therapies.

## 1. Introduction

Multiple interacting brain electrical oscillatory rhythms exist in the brain (typical frequency bands include δ: 0.5–4 Hz, θ: 4–7 Hz, α: 8–13 Hz, β: 14–30 Hz, and γ: > 30 Hz) [1,2]. These rhythms play key roles in perception, attention, memory, and motor control [3]. Abnormalities of such rhythms are closely associated with neurological and psychiatric disorders such as epilepsy, Parkinson’s disease [4], depression, and Alzheimer’s disease [5,6]. Selectively modulating specific frequency bands through external stimulation helps elucidate brain-function mechanisms and offers non-pharmacological therapeutic options [7,8]. These frequency-specific neural rhythms are commonly identified and categorized using electrophysiological recordings, particularly electroencephalography (EEG), where canonical bands such as δ, θ, α, β, and γ are routinely used to describe brain states and dysfunctions [9,10]. From an engineering perspective, this also highlights the need for stimulation hardware that can reliably generate programmable rhythmic outputs matched to distinct oscillatory bands.

In recent years, neuromodulation techniques based on amplitude modulation (AM) have become an emerging approach for stimulating specific brain rhythms. Combining transcranial alternating-current stimulation (tACS) with AM yields AM-tACS, one of the most commonly used methods. Omae et al. controlled an electrical stimulator to generate AM waveforms with a 120 Hz carrier and 15 Hz envelope to stimulate the β band in post-stroke aphasia patients. Both single-session and repeated interventions (10 sessions) improved naming accuracy and language performance [11]. Haslacher et al. used Simulink to generate voltage signals that were amplified into AM waveforms (carrier: 40 Hz, envelope: 6 Hz), demonstrating the feasibility of AM-tACS in enhancing or suppressing specific brain oscillations [12]. One year later, Haslacher et al. generated 8 kHz carriers modulated by 8–14 Hz envelopes to regulate α waves, improving working-memory accuracy and demonstrating the advantages and clinical potential of AM-based neuromodulation [13]. Another AM-based approach is temporal interference stimulation (TIS), which produces an AM envelope from the difference between two high-frequency currents. Grossman et al. applied 2.00 kHz and 2.01 kHz sine wave currents via scalp electrodes in mice and observed neuronal responses following the interference envelope [14]. Violante et al. applied 2.00 kHz and 2.005 kHz carriers (5 Hz difference) to the human scalp; through electric-field modeling and cadaver measurements, they confirmed that TIS can focus precisely on the hippocampal region while reducing cortical stimulation. Combined fMRI and behavioral tests showed that TIS specifically modulates hippocampal activity and improves episodic-memory accuracy [15]. Wang et al. applied 2.00 kHz and 2.001 kHz difference-frequency stimuli (1 Hz envelope) to mice and found, via calcium imaging and eye-movement tracking, that TIS effectively modulates superior-colliculus neural activity and evokes ocular motion [16].

Currently, transcranial rhythmic electrical stimulation typically uses scalp electrodes to deliver AC currents with specific envelope or difference frequencies. However, due to the irregular conductivity of cranial tissues and the high resistivity of the skull, electrical stimulation suffers from weak spatial focality and limited depth, usually confined to the cortex [17]. Although high-frequency carriers (as in high-frequency tACS and TIS) partially reduce skull impedance and improve depth, they still remain insufficient for precise, high-resolution deep-brain modulation [18]. Transcranial magneto-acoustic stimulation (TMAS) is an emerging neuromodulation technique that utilizes focused ultrasound directed into tissue placed within a static magnetic field. Based on the magneto-acoustic effect, focused electric fields are induced inside biological tissue, enabling noninvasive, spatially focused electrical stimulation in deep-brain regions [19]. Experiments by Wang [20], Zhang [21], Chu [22], and others have shown that TMAS interventions in PD/AD animal models yield positive behavioral and electrophysiological outcomes. Therefore, using TMAS for rhythmic stimulation can directly and precisely modulate deep-brain neural oscillations [23].

To achieve rhythmic stimulation of deep-brain regions using TMAS, the generated AM waveform must satisfy several requirements: a high ultrasound carrier frequency (200–650 kHz) to ensure sufficient penetration and focusing [24], and a very low envelope frequency (e.g., 0.5–4 Hz δ band) to match physiological rhythms. Liu et al. previously applied a difference-frequency approach to TMAS using 500 kHz and 505 kHz carriers to obtain a 5 kHz beat envelope, but such methods cannot achieve the large carrier–envelope frequency separations required for rhythmic stimulation [25]. Importantly, the carrier–envelope separation addresses the temporal requirement of rhythmic stimulation, whereas the 64-channel phased-array addresses the spatial requirement of controllable focusing/steering. Accordingly, the multi-channel design is selected to provide phase-coherent array driving rather than to realize AM separation per se. Moreover, TMAS phased-array systems demand precise multi-channel outputs to drive phased-array transducers for accurate focusing, whereas conventional software-controlled signal generators are ill-suited for multi-channel expansion and precise phase alignment. Finally, the system must maintain reliability and ease of use while balancing temporal–frequency selectivity and channel synchronization.

Despite the progress of rhythm-specific neuromodulation, several research gaps still remain. First, most existing frequency-specific stimulation approaches are based on transcranial electrical stimulation, which still faces limitations in stimulation depth and focality. Second, although TMAS offers the inherent advantages of high-frequency penetration and focal control, there is still a lack of a multi-channel AM-TMAS driving system that can conveniently generate AM waveforms featuring high carrier frequencies and low-frequency envelopes corresponding to brain rhythms. Third, the engineering validation of waveform fidelity, multi-channel synchronization, and rhythmically modulated magneto-acoustic electrical responses remains limited.

To address these gaps, the major contributions of this study are as follows:

1. TMAS technology is combined with AM to design and implement a multi-channel AM-TMAS driving system based on phased arrays, enabling a single device to control multiple channels and generate AM waveforms with high carrier frequencies and low-frequency envelopes corresponding to brain rhythms.
2. A DDS/LUT-based digital synthesis method is implemented on an FPGA + high-speed DAC platform, allowing the system to maintain the inherent advantages of high-frequency penetration and focal control while delivering controllable stimulation at specific rhythmic frequency bands.
3. The proposed system is validated through electrical output measurements and magneto-acoustic electrical experiments in a saline medium under a static magnetic field, confirming waveform fidelity, multi-channel synchronization, and rhythmic modulation characteristics consistent with the stimulation envelope.

The rest of this paper is organized as follows. Section 2 presents the principle of TMAS and the design of the multi-channel AM-TMAS driving system. Section 3 describes the experimental setup and results. Section 4 discusses the significance of this work and future development directions. Section 5 concludes the paper.

## 2. Materials and Methods

### 2.1. Basic Principle of Tmas

The core mechanism of transcranial magneto-acoustic stimulation (TMAS) lies in magneto-acoustic coupling between an acoustic field and an externally applied magnetic field [26]. In essence, ultrasound-induced microscopic motion of charged particles within biological tissue, when exposed to a static magnetic field, generates controllable electric fields or currents that directly modulate neuronal membrane potentials [27]. From the perspective of electromechanics, the phenomenon can be described quantitatively. Let the instantaneous acoustic pressure be p. In an approximately plane or locally uniform acoustic field, acoustic impedance Z (where Z = ρc, with ρ being medium density and c the sound velocity) relates pressure to particle velocity **v** by:

(1) $$v(r,t)\approx \frac{p(r,t)}{Z}\hat{n},$$

where **n̂** denotes the unit vector along the acoustic propagation direction. If the equivalent charge density of mobile ions is ρ_e_, the current density produced by these moving charges is:

(2) $$J(r,t)=\rho_{e}(r)\;v(r,t)$$

When this motion occurs in an externally applied static magnetic field B, the moving charges experience a Lorentz force, which leads to an induced motional electromotive field approximately expressed as:

(3) Eind(r,t)≈(p(r,t)Zn^)×B

From Equations (2) and (3), in conductive biological tissue placed within a static magnetic field, an incident ultrasound wave induces an internal electric field and current perpendicular to both the acoustic propagation direction and the magnetic field. The induced electric-field distribution follows the spatial pattern of the acoustic field, and for given B and Z, the local field amplitude is proportional to acoustic pressure p. By designing the spatial distribution of the acoustic field (e.g., via multi-channel phased focusing) and adjusting the direction and intensity of B, it is possible to generate controllable, focal electric fields at the target region—thereby achieving localized deep-brain electrical stimulation [28].

### 2.2. Am Hardware Architecture of the Am Module

**Figure 1:**
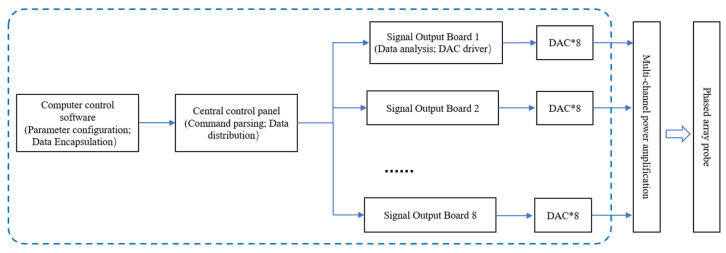
Hardware architecture of the AM module.

The overall architecture of the AM module is shown schematically in Figure 1 (blue box region). It consists of three hierarchical layers: Host PC → Central Control Board → Signal-Output Boards, capable of generating and controlling 64 arbitrary analog output channels. The host PC is responsible for experiment configuration, waveform and envelope generation and transmission, start/stop control, and status feedback. The central control board parses commands, distributes parameters to submodules, and manages communication and board states. Each signal-output board performs on-board AM-envelope computation and waveform synthesis, driving high-precision analog outputs through on-board DACs. The generated 64-channel AM signals are then amplified by multi-channel linear power amplifiers to reach the voltage and power levels required to excite the phased-array transducer elements, forming the desired focused acoustic field [29,30].

After the host computer transmits control and waveform data to the central control board, the central control board distributes the signals to eight signal-output boards [31]. Each output board carries four dual-channel DACs (eight channels per board), forming a total of 8 × 8 = 64 channels in the AM signal-generation module. Each signal-output board uses an Altera Cyclone IV EP4CE75 FPGA (Altera, San Jose, CA, USA) as the main controller. This FPGA provides ample logic and memory resources for waveform storage and protocol parsing, enabling high-speed, large-volume waveform storage and distribution [32]. Its abundant computational resources also facilitate real-time envelope calculation [33]. The FPGA’s built-in PLL supports multi-rate, high-precision clock generation, allowing the system to operate with a 100 MHz master clock while supplying a 125 MHz high-speed clock to the DACs—further improving waveform accuracy [34]. Moreover, the FPGA’s rich I/O interfaces support simultaneous multi-channel output. To enhance software resource reuse and improve development efficiency, the central control board and the signal-output boards share the same FPGA model. The chosen DAC device is the AD9767 (Dual 14-bit, 125 MSPS; Analog Devices, Wilmington, MA, USA). Its 14-bit resolution and 125 MSPS update rate meet the requirements for high-amplitude precision and wide carrier bandwidth. The DAC’s differential current outputs and simultaneous update (UPDATE/WRT) capability make it well-suited for precise multi-channel synchronized output. This hardware architecture enables arbitrary waveform generation and the focusing of control across 64 channels. With its abundant hardware resources, the system can perform board-level AM algorithm computations, allowing the DACs to generate high-speed, high-precision waveforms that fully meet the performance demands of AM-TMAS neuromodulation experiments [35].

### 2.3. Software Design of the Am Module

**Figure 2:**
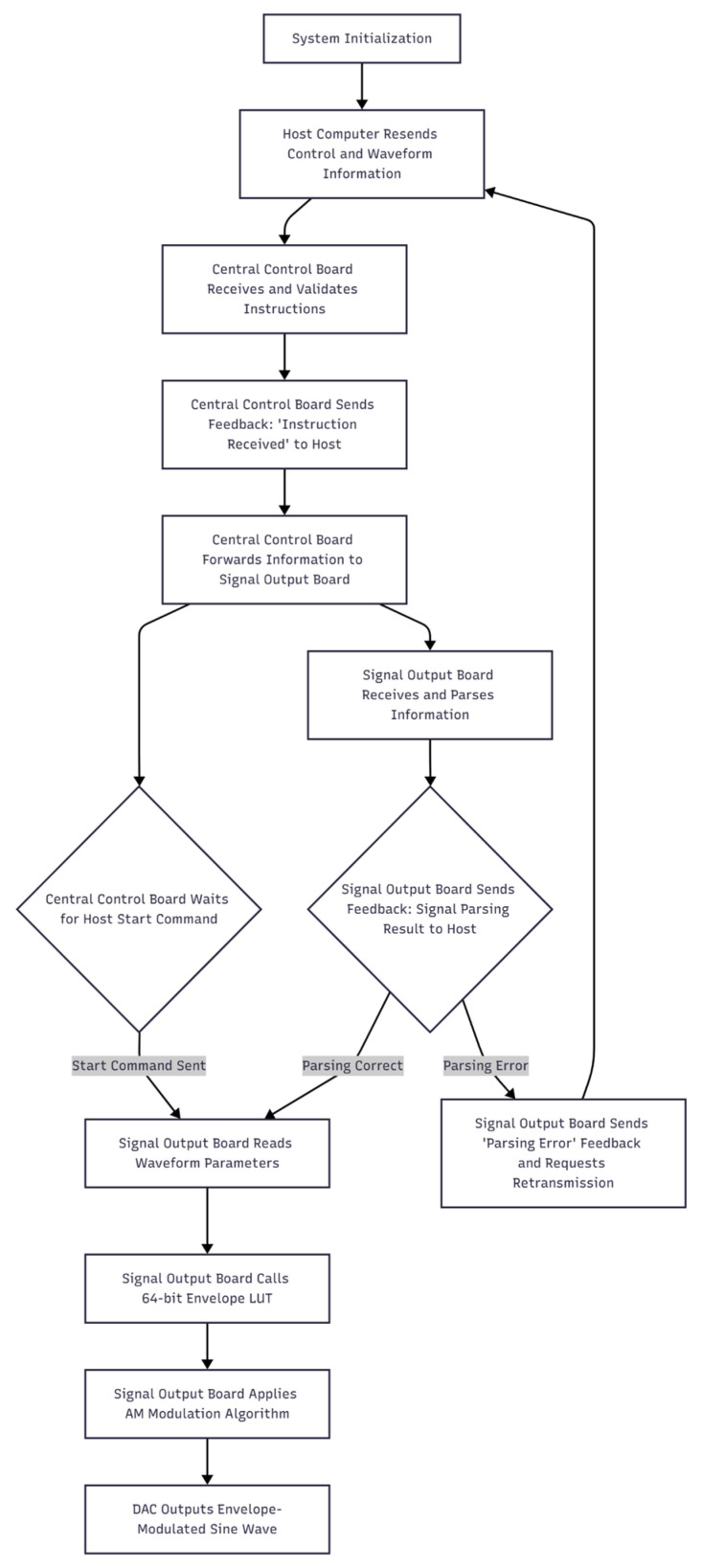
Software architecture of the AM module.

As shown in the AM module software flowchart (Figure 2), the host computer transmits control and waveform information to the central control board, which then distributes the data to the signal-output boards. The signal-output boards perform envelope computation and output the resulting waveforms. The host (PC) is responsible for configuring system parameters and waveform data. It packages experimental protocols and waveform information into communication frames, issues start/stop/trigger commands, and it receives the system’s operational status as well as logs. Communication between the host and the central control board is implemented via a serial interface. After sending a command, the host waits for an acknowledgment from the control board. In addition, the host manages waveform batches, records timestamps, and stores operation logs to facilitate subsequent reproducibility checks and software calibration. This frame format, together with its acknowledgment mechanism, enables the host software to implement framing, retransmission, integrity checking, and result logging, ensuring reliable and verifiable data communication throughout the AM module control process. The host–FPGA control interface uses structured configuration with parameter validation (e.g., carrier/envelope frequency, amplitude/modulation depth, and channel enable) before changes are applied, and waveform updates take effect only on the global synchronous UPDATE event to avoid asynchronous transients. In addition, host-side operation logging provides traceability of stimulation configurations for controlled laboratory use.

After system initialization, the host computer transmits waveform parameters, trigger intervals, and runtime data to the central control board via a serial interface. Upon receiving this information, the central control board performs syntax and consistency checks, then preloads the key runtime parameters to the signal-output boards and stores them in on-chip memory. Preloading complex waveform parameters before operation significantly simplifies the real-time control logic—during runtime, the system only needs to handle lightweight control events (such as start, stop, and trigger), which reduces timing complexity and improves overall system stability. During the startup phase, the system transitions from static configuration to periodic operation. When a start command is received, the central control board simultaneously issues a one-time start pulse to the designated sub-boards and activates a periodic retrigger module to generate global UPDATE signals at fixed intervals. The global UPDATE signal is broadcast to all sub-boards through the backplane distribution network so that envelope/carrier updates take effect synchronously across channels. The initial start pulse provides all subsystems with a unified time reference, while the subsequent retriggers prevent drift and cumulative jitter in software timing, and mitigate the risks of race conditions and metastability in hardware state machines. Afterward, each signal-output board retrieves the corresponding envelope data, applies the AM algorithm to synthesize the waveform, and drives the DAC to output amplitude-modulated signals with high-precision envelopes, thereby achieving accurate phase-controlled focusing. This software architecture establishes a configurable, verifiable, and highly reliable multi-channel control and data transmission system on the FPGA platform, laying the groundwork for the efficient hardware implementation of the AM algorithm.

### 2.4. Implementation of Amplitude-Modulated Sine Waves

#### 2.4.1. AM Algorithm

Amplitude modulation (AM) employs a slowly varying envelope function to control the amplitude of a high-frequency carrier. Its most common mathematical representation is:

(4) s(t)=[1+m(t)]Ac  cos(ωc t+ϕ),

where Ac is the carrier amplitude, ωc is the carrier angular frequency, ϕ is the phase, and m(t) is the normalized envelope [36]. By equivalent transformation, the equation can also be written as:

(5) s(t)=E(t)cos(ωc t+ϕ)

where $E(t)=[1+m(t)]A_{c}$. This formulation highlights two key points: The carrier c(t) determines penetration depth and frequency selectivity; the envelope E(t) governs the low-frequency rhythmic modulation (e.g., θ or α bands), meaning the stimulation should match the intrinsic physiological oscillations. An ideal envelope must meet the following requirements: it should be smooth (to avoid high-frequency interference caused by abrupt changes), have a configurable spectrum (matching the target neural rhythm), and maintain amplitude accuracy. The discrete waveform generated by the DAC must also satisfy these conditions [37].

To implement amplitude-modulated waveforms on an FPGA, the continuous-time model must be discretized. Discretization involves two signal types: the carrier sequence x (read from memory at sampling rate $f_{s}$) and the envelope sequence E. In the discrete domain, the amplitude-modulated waveform can be expressed as:

(6) y[n,k]=E[k]⋅x[n],

where n indexes the carrier samples and k indexes the envelope samples. Most DACs and storage formats use unsigned representation (e.g., 16-bit unsigned). If the envelope coefficients are multiplied directly in the unsigned domain, asymmetric truncation occurs near zero or maximum amplitude, causing phase shifts or waveform distortion during amplitude attenuation. To ensure the modulation occurs within a symmetric numerical domain, the unsigned samples x must first be mapped to a signed range centered at zero. Let $A_{0}$ denote the midscale bias (typically half the numeric range). Then, we have the following:

(7) $$x_{s}[n]=x[n]-A_{0}.$$

After scaling in the signed domain, the bias is added back. This “center–multiply–restore” procedure allows multiplication to be performed symmetrically about zero, eliminating phase offset caused by unsigned truncation and preserving waveform symmetry across positive and negative half-cycles. If the envelope coefficients E are represented as fixed-point integers with radix Q (e.g., Q = 2^16^), the digital implementation becomes:

(8) $$z[n,k]=\frac{x_{s}[n]\cdot E[k]}{Q}.$$

and the final DAC output is:

(9) $$y[n,k]=\mathrm{sat}\left(z[n,k]+A_{0}\right),$$

where sat (⋅) denotes saturation clipping within the DAC output range to prevent overflow. Equations (8)–(9) represent the core design principle of the AM module: first center, then multiply by the fixed-point envelope, and finally restore the bias. This method offers three major advantages: Multiplication occurs in a signed domain, maintaining strict waveform symmetry; fixed-point arithmetic eliminates the computational burden of floating-point operations, improving processing speed; fixed-point multiplication and LUT access can be performed in parallel on FPGA hardware with deterministic timing, ensuring precise multi-channel synchronization.

In summary, Equations (4)–(9) define the discrete AM synthesis and the signed-domain fixed-point scaling used in this work. The next section focuses on the board-level realization, including buffering, clocking, and global UPDATE timing for implementing this synthesis in hardware.

#### 2.4.2. Implementation of the AM Algorithm on the Signal-Output Board

Each signal-output board converts control commands and preloaded waveform parameters into multi-channel, synchronous, and envelope-shaped AM sine stimulation signals. To reconcile experimental flexibility with real-time performance, the system employs a hierarchical data-management architecture: after receiving control frames, the signal-output board writes control parameters and waveform descriptors into a main storage area, then uses a DMA-like(Direct Memory Access, DMA) copy module to distribute frame data into per-channel local buffer memory. Functionally equivalent to DMA, this distribution mechanism is intended to copy and buffer large waveform datasets without occupying the main controller’s execution time, allowing each channel to read its data independently and continuously for real-time output.

High-resolution carrier samples are stored in RAM on the output board, while smooth, low-frequency envelope shapes are stored in lookup tables (LUTs) [38]. Keeping carrier samples in RAM enables support for arbitrary waveform shapes and arbitrary lengths of carrier fragments (for example, non-sinusoidal carriers, experiment-specific segments, or long periodic sequences), thus increasing carrier flexibility; storing envelopes in LUTs minimizes resource usage while enabling rapid retrieval of envelope coefficients. At runtime, each channel reads carrier samples from its local RAM buffer according to an index pointer and concurrently fetches the current envelope coefficient from the LUT according to a predefined envelope-stepping strategy. The envelope LUT length and update interval are configurable in our implementation; the 64-entry envelope table used in this work is a resource-efficient setting for the reported validation cases. For envelopes at the extreme low-frequency bound, higher LUT resolution and/or linear interpolation between LUT points can be enabled to reduce zero-order-hold staircasing and the associated spurious modulation components. The amplitude scaling (multiplication) is performed in hardware, and the scaled samples are forwarded to the DAC. After each carrier cycle is completed, the envelope index advances according to the schedule, producing a slowly varying amplitude envelope across many high-frequency cycles. This beat-coupling approach enables precise control of envelope frequency while maintaining timing stability. Using this method, sine AM signals with envelope frequencies adjustable from 0.1 Hz to 100 kHz and carrier (fundamental) frequencies adjustable from 100 kHz to 2 MHz can be generated.

To achieve deterministic, synchronous multi-channel output, the system applies an optimized clocking and update strategy. In this manuscript, “high-precision” refers to a bounded inter-channel timing skew and verified carrier/envelope frequency accuracy, as quantified in the Results. The module logic and the DAC channels use separate clocks—the module uses a lower-frequency clock to stabilize control and peripheral timing and avoid interfering with DAC operation; all DACs share a dedicated, high-frequency, low-jitter clock, and a global synchronous trigger signal is used to effect simultaneous data updates. Waveform data in the module are prepared and verified in the local buffers for the next frame; when ready, the control logic issues a global UPDATE signal so that all output channels present their new data to the DACs on the same trigger edge, minimizing inter-channel timing skew [39]. Cross-clock-domain data paths use synchronized FIFOs to prevent metastability; critical clock paths are constrained during simulation and verified by static timing analysis to ensure the module operates stably.

The AM generation scheme presented and implemented in this section—DDS + LUT + envelope multiplication—is engineered with half-wave LUT envelope storage, DMA-style data transfers, a precise and efficient AM algorithm, and a global UPDATE synchronization mechanism. Together, these measures guarantee 14-bit resolution, high accuracy, and real-time performance. The implementation supports envelope tunability from 0.1 Hz to 100 kHz, carrier tunability from 100 kHz to 2 MHz, and adjustable envelope repetition rates, providing stable and reliable waveforms for subsequent experiments.

### 2.5. System Testing Methods

To validate the functionality and performance of the proposed multi-channel TMAS driver based on amplitude-modulated sine waves, we performed two classes of tests: multi-channel AM signal-output tests and AM-TMAS rhythmic magneto-acoustic electrical stimulation tests. The objectives were: (1) to verify that the system can generate high-fidelity amplitude-modulated sine waves; and (2) to determine whether the system can elicit induced electric fields in a physiological surrogate medium consistent with theoretical predictions.

#### 2.5.1. Modulated Signal-Output Test

**Figure 3:**
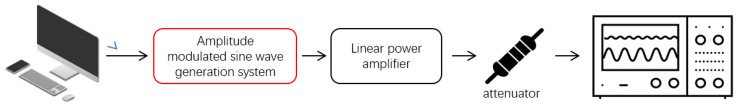
Block diagram of the modulated signal-output test.

The test used the configuration shown in the Figure 3: the AM module’s outputs were connected to multi-channel linear power amplifiers to form a multi-channel TMAS system based on amplitude-modulated sine waves. The amplifier outputs passed through a power attenuator and were then recorded by RIGOL DS7024 digital oscilloscope (RIGOL Technologies, Suzhou, China). Typical α- and γ-band envelope frequencies (8 Hz and 40 Hz, respectively) were selected, with an ultrasound carrier f = 500 kHz, to obtain the AM waveform at an arbitrary channel. Envelopes were extracted by computing the analytic signal via the Hilbert transform, applying a 4th-order Butterworth low-pass filter with a 50 Hz cutoff, and finally performing Gaussian smoothing to obtain a precise envelope estimate. We then evaluated extreme operating points of the system. To test very low envelope frequency performance, a 1 Hz envelope with a 500 kHz carrier was used; to test high carrier-frequency performance, a 40 Hz envelope with a 1 MHz carrier was employed. Finally, the multi-channel synchronization performance was evaluated: the initial delays were all set to zero, and 64 channels were sequentially tested, with the actual delay values measured using an oscilloscope (RIGOL DS7024).

#### 2.5.2. Magneto-Acoustic Electrical Measurement of the AM-TMAS System

**Figure 4:**
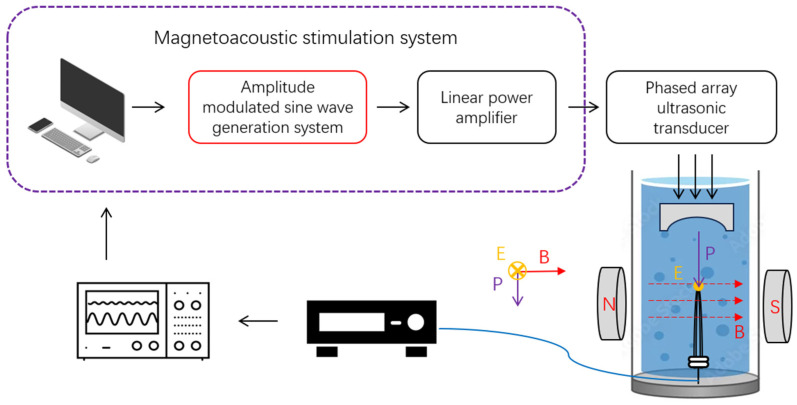
Block diagram of the AM-TMAS magneto-acoustic electrical test. Red arrows: static magnetic field (B). Purple arrows: acoustic pressure (P). Orange arrows: induced electric field (E). Blue shading: liquid medium. Yellow dot: focal point.

After confirming the front-end AM output performance, the AM-TMAS magneto-acoustic electrical measurements were carried out to evaluate whether the system can elicit the expected AM magneto-acoustic coupling/stimulation fields in a static magnetic environment. The test configuration is shown in Figure 4. The in-house AM module and custom linear power amplifiers formed the multi-channel TMAS driver, which was connected to HP-500k-64 phased-array ultrasound transducer (Doppler Electronic Technologies, Guangzhou, China). The transducer and the custom measurement electrodes were immersed together in a container filled with physiological saline (σ = 4 S/m) to emulate the electrical properties of neural tissue. A permanent magnet providing a stable magnetic field was placed adjacent to the container; the magnetic flux density was measured with Model 410 gaussmeter(Lake Shore Cryotronics, Inc., Westerville, OH, USA) and found to be 0.3T. During detection, the focused ultrasound propagation direction was kept orthogonal to the magnetic field so that the magneto-acoustic-induced field arose along the vector cross-product direction of the acoustic velocity and the magnetic field.

Test and sampling parameters for the AM magneto-acoustic measurement were: excitation carrier: 500 kHz; envelope: 40 Hz; and data acquisition sampling rate: 10 MHz. Raw waveforms were recorded while the FPGA/host maintained timestamps and trigger markers to ensure time alignment. The electrode-captured magneto-acoustic signal was preamplified by OLYMPUS KN010327 preamplifier (Olympus Corporation, Tokyo, Japan) with 60 dB gain and input to the RIGOL DS7024 oscilloscope. Because the magneto-acoustic signals had a low signal-to-noise ratio, the oscilloscope recorded averaged waveforms using 8× averaging. The envelope extraction of the AM magneto-acoustic signal follows the same procedure as described in the previous subsection.

## 3. Results

### 3.1. Multi-Channel AM Signal-Output Tests

**Figure 5:**
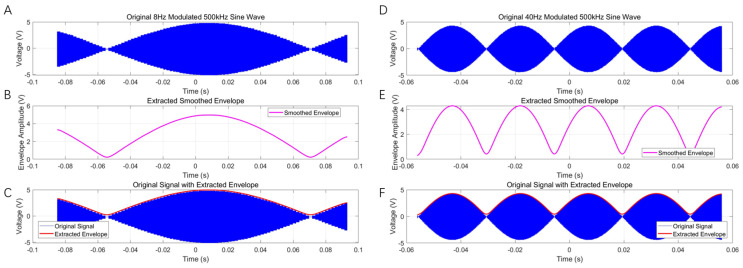
AM sine waveforms from an arbitrary channel with 8 Hz and 40 Hz envelopes (**A**,**D**); the carrier frequency in both cases is 500 kHz. (**B**,**E**) present the extracted envelopes. (**C**,**F**) present the extracted envelopes overlaid on the raw signals for 8 Hz and 40 Hz, respectively.

Figure 5 presents the AM sine waveforms with 8 Hz and 40 Hz envelope output from a representative channel, both with a carrier frequency of 500 kHz. Figure 5B,E show the extracted envelopes obtained by applying the Hilbert transform, followed by low-pass filtering and Gaussian smoothing. The extracted envelope frequencies are clearly identified as 8 Hz and 40 Hz, respectively. Figure 5C,F illustrate the superposition of the extracted envelopes onto the original signals, demonstrating that the extracted envelopes accurately match and fully cover the original waveforms. To quantify this agreement, we computed the normalized root mean square error (NRMSE) between the extracted envelope and the intended envelope. The NRMSE is 1.0795% for the 8 Hz case and 1.9212% for the 40 Hz case (Figure 5). As shown in Figure 5, the system successfully generates composite waveforms in which low-frequency envelopes (8 Hz and 40 Hz) are superimposed on high-frequency carriers (500 kHz). The envelopes vary smoothly without noticeable distortion, confirming that the amplitude modulation algorithm based on DDS + lookup table + envelope multiplication has been correctly implemented and operates with high fidelity.

**Figure 6:**
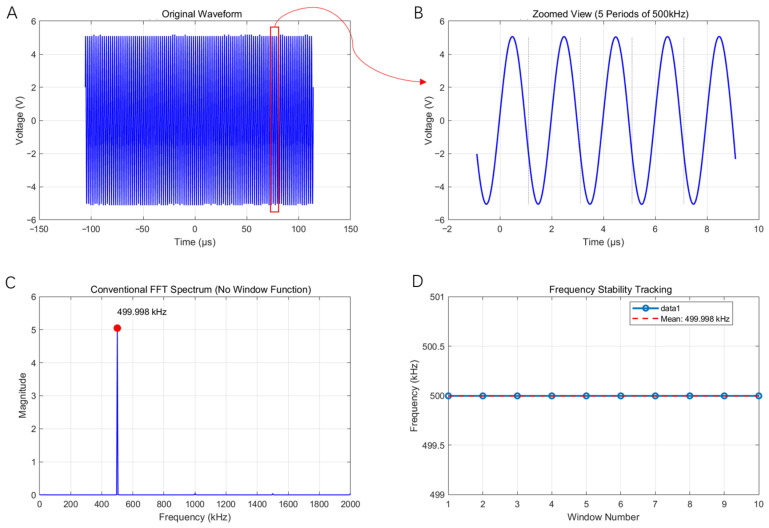
The carrier (fundamental) output tests for the AM signal. (**A**) A segment of the signal within a 40 Hz envelope; (**B**) a zoomed view of (**A**); (**C**) amplitude spectrum of (**A**), with energy concentrated at 499.998 kHz; (**D**) carrier frequency stability over time.

To further verify the carrier accuracy, we extracted and analyzed a time segment of the recorded signal under the 40 Hz envelope. Figure 6A,B demonstrate the zoomed, extracted segment and indicate a smooth, undistorted carrier waveform. Figure 6C shows the spectrum of the carrier, peaking at 499.998 kHz. For frequency-stability testing (Figure 6D), we sampled the time series every 50 μs and computed the spectrum for each 20 μs window; each window’s dominant frequency was 499.998 kHz, demonstrating excellent carrier stability.

Overall, comparison of the 8 Hz and 40 Hz envelope cases shows that the system accurately reproduces the intended amplitude modulation characteristics at different rhythmic frequencies. The 40 Hz envelope has a shorter period (25 ms) with smooth oscillations and clear peaks/troughs; the 8 Hz envelope has a longer period (125 ms) and a slower amplitude modulation. The spectral analysis and stability tests confirm that the 500 kHz carrier was generated correctly.

**Figure 7:**
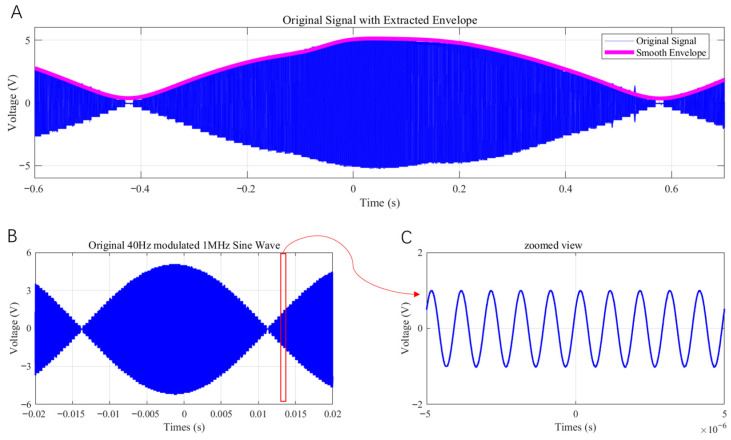
(**A**) Sine wave AM signal with a 500 kHz carrier and a 1 Hz envelope. (**B**) AM signal with a 1 MHz carrier and a 40 Hz envelope. (**C**) Magnified view of panel (**B**).

To further validate the flexibility of the multi-channel AM module’s output, Figure 7A shows the waveform from an arbitrary channel with a 500 kHz carrier and a 1 Hz envelope. Even at this very low envelope frequency, the system reliably produces an accurate AM waveform, demonstrating that the implemented AM algorithm handles extremely large carrier–envelope frequency separations effectively; the system can therefore generate AM stimulation waveforms with envelope frequencies within the δ band (0.5–4 Hz). Figure 7B,C show the waveform from an arbitrary channel with a 1 MHz carrier and a 40 Hz envelope (C is a magnified view). When the carrier frequency is increased, the AM-generation module based on the described algorithm still produces smooth carrier oscillations with clearly defined envelopes, confirming robust performance across higher carrier frequencies.

**Figure 8:**
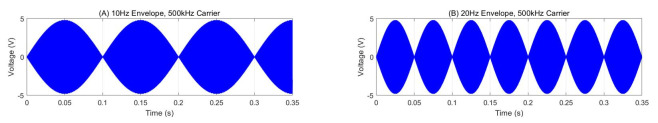
AM sine waveforms from an arbitrary channel with 10 Hz and 20 Hz envelopes, corresponding to the α and β bands, respectively; the carrier frequency in both cases is 500 kHz.

To further validate the flexibility of the multi-channel AM module’s output across representative brain electrical oscillatory rhythms, additional waveforms with a 500 kHz carrier and 10 Hz and 20 Hz envelopes were generated in Figure 8, corresponding to the α and β bands, respectively. Combined with the 8 Hz and 40 Hz cases in Figure 5, representing the θ and γ bands, and the 1 Hz case in Figure 7A, corresponding to the δ band, these results demonstrate that the proposed system can generate AM stimulation waveforms covering the five representative brain-rhythm bands: δ, θ, α, β, and γ.

**Figure 9:**
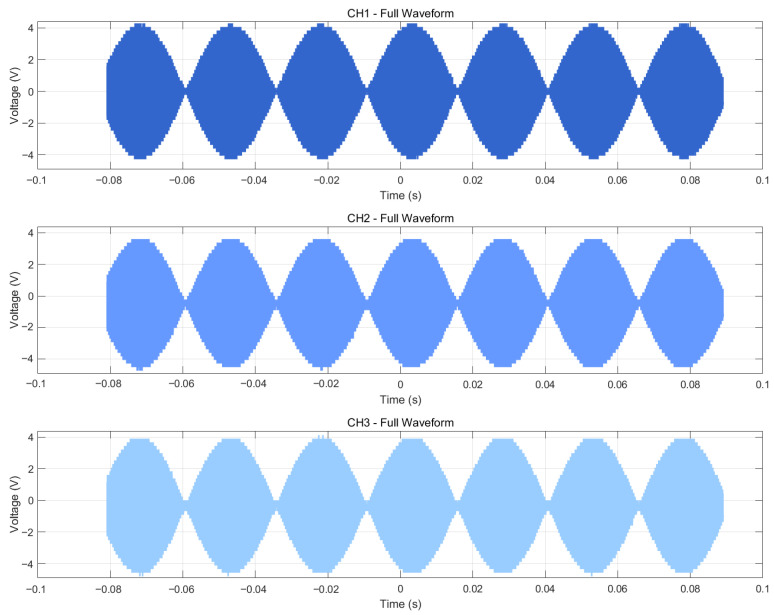
AM waveform output from any three channels.

Figure 9 displays AM waveforms from three arbitrarily selected channels (CH1, CH2, and CH3). The three channels are highly consistent in amplitude and phase; their output waveforms closely match one another, demonstrating that the FPGA–DAC parallel update strategy effectively achieves synchronous multi-channel output.

**Figure 10:**
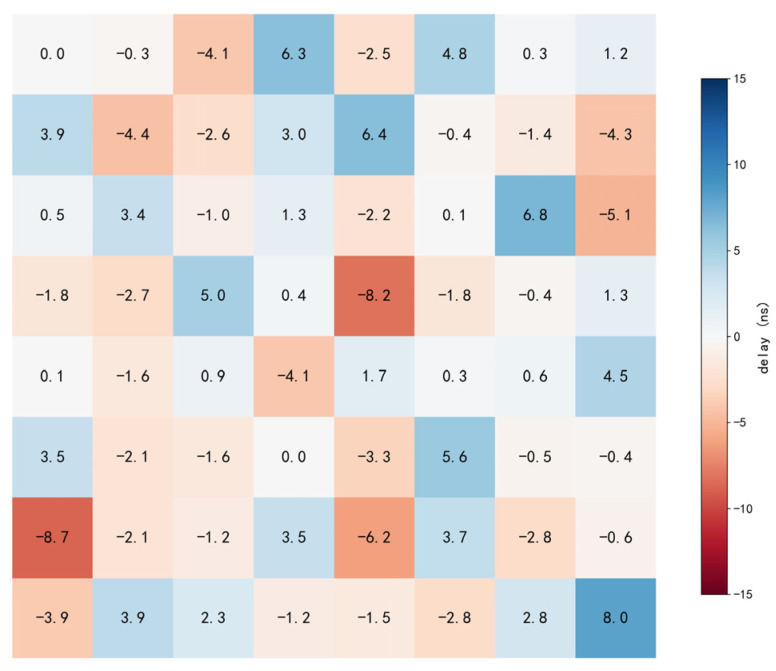
64-channel delay test.

Figure 10 shows the full 64-channel delay map (8 × 8). Delays are measured at the outputs relative to a reference channel with a shared clock and global UPDATE, so the values are the end-to-end inter-channel skew and already include distribution-network propagation mismatch.

With the dataset, the skew distribution remains bounded across the array and is centered close to zero: the mean relative delay is 0.76 ns (variance 14.90 ns^2^, SD 3.86 ns). The measured values span approximately −8.0 to +8.7 ns, corresponding to a peak-to-peak skew of 16.7 ns across the full 64-channel array. For the nominal carrier frequency used in this work (500 kHz), this peak-to-peak timing spread corresponds to an upper-bound phase spread of approximately 3.0° across channels (Δφ = 360°·Δt/T). This full-array result therefore provides a direct and quantitative bound on deterministic inter-channel skew for phase-coherent 64-channel AM driving; if tighter phase matching is required for a specific array integration, the residual bounded skew can be further reduced through per-channel phase/delay trimming while preserving the same global clock/UPDATE synchronization framework.

In summary, the multi-channel AM module output tests validate that the proposed FPGA + DAC hardware architecture, together with the DDS + LUT + envelope-multiplication AM algorithm, can generate high-fidelity amplitude-modulated sine waves: high-frequency carriers are reliably combined with low-frequency envelopes, providing a stable and dependable drive signal foundation for subsequent magneto-acoustic-coupling stimulation experiments.

### 3.2. AM Magneto-Acoustic-Coupled Electrical Signal Test

**Figure 11:**
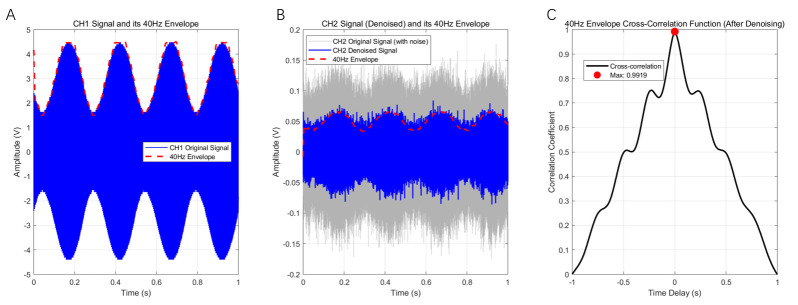
Results of the magneto-acoustic-coupled electrical signal test. (**A**) AM module output signal after eightfold averaging; the red line represents the extracted low-frequency envelope. (**B**) Raw AM magneto-acoustic-coupled electrical signal after eightfold averaging (gray), the filtered AM magneto-acoustic signal (blue), and the extracted low-frequency envelope (red). (**C**) Time-domain cross-correlation curve of the envelopes from signals (**A**,**B**), with a peak correlation coefficient = 0.9919.

Figure 11 presents the results of the magneto-acoustic-coupling electrical signal test. Panel (A) shows the AM module’s output signal after eightfold averaging, with the red line indicating the extracted low-frequency envelope obtained using the Hilbert transform combined with low-pass smoothing. Panel (B) displays the raw AM magneto-acoustic-coupled electrical signal after eightfold averaging (gray trace). After a 10 kHz cutoff Butterworth low-pass filter was applied to remove high-frequency noise, and a 5-point median filter was used to eliminate pulse noise and outliers, the resulting waveform (blue line) was obtained. Eightfold averaging and a 5-point median filter are applied due to the high ambient noise floor in magneto-acoustic response acquisition, improving SNR for envelope extraction. The red line shows the low-frequency envelope extracted from the filtered signal using the same Hilbert-based method. From panels (A) and (B), it can be seen that although both the AM output and the magneto-acoustic signal exhibit waveform changes compared to Figure 5 and Figure 8—owing to the averaging process—the envelopes remain highly consistent. Comparing the time-domain signals before and after denoising in panel (B), it is evident that low-pass and median filtering effectively remove high-frequency and impulse noise, significantly improving the envelope detection accuracy and increasing the peak value of the correlation. Panel (C) shows the time-domain cross-correlation coefficient between the two extracted envelopes from (A) and (B), yielding a peak correlation of 0.9919. The high cross-correlation indicates that the envelopes of the two signals are almost perfectly synchronized, demonstrating that under the given experimental conditions, the system successfully generated magneto-acoustic-coupled electrical signals consistent with the excitation waveform.

## 4. Discussion

This study designed and validated a multi-channel TMAS (transcranial magneto-acoustic stimulation) system based on amplitude-modulated (AM) sine waves. The system can generate 64-channel AM signals with envelope frequencies tunable from 0.1 Hz to 100 kHz and carrier frequencies from 100 kHz to 2 MHz, while meeting multi-channel phase-control requirements. In magneto-acoustic-coupling tests conducted in saline under a static magnetic field, the system successfully produced magneto-acoustic electrical responses corresponding to a 500 kHz carrier and 40 Hz envelope.

The main purpose of this study is to explore a method for generating rhythmic electrical stimulation in deep-brain regions. Conventional transcranial ultrasound stimulation modulates neural activity through mechanical effects, whereas TMAS is intended to produce localized electrical stimulation through the magneto-acoustic-coupling effect. Brain oscillations are low-frequency electrical signals. From this perspective, AM-TMAS is more closely related to rhythm-based electrical neuromodulation methods, such as temporal interference stimulation (TIS), which modulates brain activity using electric fields with specific frequencies or envelopes. The potential value of AM-TMAS lies in its ability to combine the penetration and focusing capability of ultrasound with the modulatory properties provided by electrical stimulation, thereby enabling more precise and effective neuromodulation. This may have potential applications in the study of diseases associated with abnormal neural rhythms, such as Parkinson’s disease and epilepsy [40,41].

Compared with the difference-frequency method, the proposed multi-channel AM generation and control module offers three major advantages: Enhanced programmability: the digital DDS + LUT + envelope-multiplication architecture allows fine, software-level control of envelope frequency, modulation depth, and phase, whereas the difference-frequency approach provides limited control over phase and amplitude; easier multi-channel expansion and synchronization: the unified system clock and parallel UPDATE mechanism support a large number of output channels with precise timing; and superior waveform stability and real-time tunability, facilitating subsequent biological and electrophysiological experiments.

In the TMAS field, earlier animal studies have shown that TMAS can improve memory, enhance synaptic plasticity, and exert neuroprotective effects in pathological models [42]. The present work provides an important engineering-level complement by verifying the measurability and inter-channel consistency of magneto-acoustic electrical signals—an essential prerequisite before biological intervention. Our results demonstrate that under 500 kHz/40 Hz AM conditions, the system produces highly consistent and high-fidelity stimulation signals across channels, offering a reliable hardware platform for future investigations into rhythm-specific neural modulation at different frequencies.

**Figure 12:**
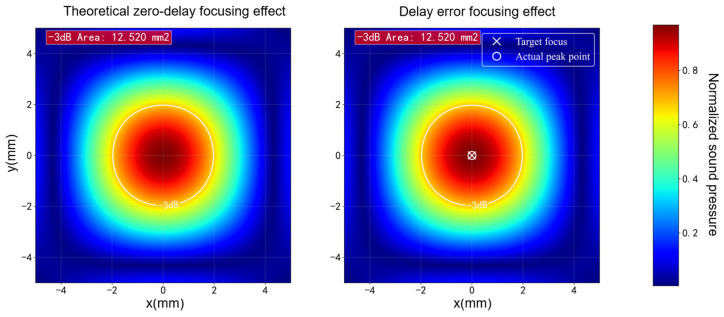
Simulated 64-channel acoustic field under (**left**) ideal phase alignment and (**right**) measured inter-channel synchronization error. The −3 dB contour is overlaid; the target focus and actual peak point are marked.

To evaluate how inter-channel delay errors affect the spatial focusing accuracy of phased-array beamforming, acoustic-field simulations were performed for the 64-channel array by comparing an ideal case with zero delay error and a perturbed case incorporating the measured inter-channel delays, as shown in Figure 12. The simulations used a planar-array transducer model consistent with the experimental setup, with a center frequency of 500 kHz, an element width of 4.3 mm, an element pitch of 4.5 mm, and a focal distance of 50 mm. Considering the trade-off between stimulation depth and spatial resolution, 500 kHz is a commonly used operating frequency for transcranial deep-brain stimulation because it enables substantial acoustic energy transmission to deep targets while maintaining practical focusing accuracy. As shown in Figure 12, introducing the measured delay errors does not change the focal-spot size, with the −3 dB focal area remaining 12.520 mm^2^, and the peak-pressure point shifting by only 0.08 mm, which is negligible relative to the approximately 3.5 mm focal-spot diameter. These results indicate that, at the 500 kHz operating frequency used here, the current level of inter-channel delay error has only a very limited effect on focusing performance.

**Figure 13:**
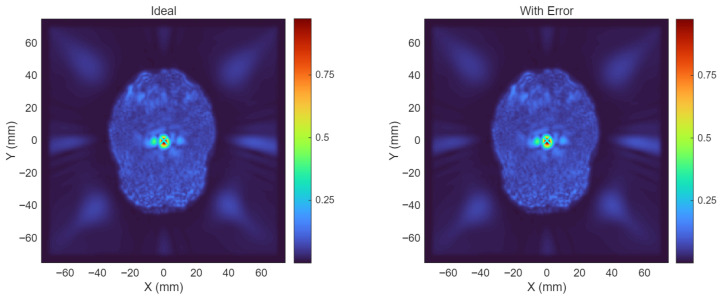
Simulated transcranial acoustic pressure fields in the skull model for the ideal case and for the case with measured inter-channel delay errors. The color bars indicate normalized acoustic pressure amplitude.

**Figure 14:**
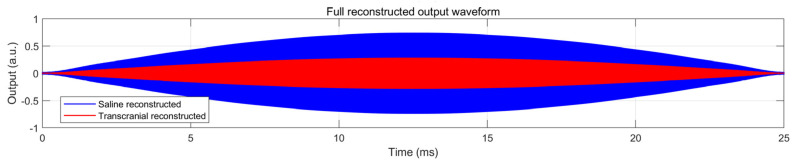
Time-domain reconstructed output waveforms in saline and under transcranial propagation through the skull model.

To further assess transcranial propagation, the acoustic field in a skull model was simulated, as shown in Figure 13. The results show that the focused region remains clearly localized after propagation through the skull. This tendency is also reflected in the reconstructed time-domain waveforms in Figure 14: compared with the saline case, the transcranial reconstructed waveform exhibits lower amplitude, but its low-frequency modulation envelope remains preserved. Taken together, Figure 12, Figure 13 and Figure 14 suggest that the proposed AM-TMAS system maintains both spatial focusing capability and rhythmic modulation characteristics under the present operating condition, while the main effect of skull transmission is attenuation rather than obvious loss of envelope structure. In future work, the system phase-delay error can be further reduced by adopting higher-performance FPGAs, and more realistic transcranial models can be used to further evaluate acoustic transmission and rhythm–envelope preservation.

Safety should also be considered under the present AM-TMAS operating conditions. For the 0.5 MHz carrier and 0.5 MPa peak-to-peak acoustic pressure used in this study, the estimated mechanical index (MI) is approximately 0.35, indicating a relatively low cavitation risk. In addition, under the 40 Hz sinusoidal amplitude-modulated stimulation condition, the estimated effective acoustic power is about 0.200 W, the equivalent aperture diameter is about 4.06 cm, and the corresponding cranial thermal index (TIC) is approximately 0.62. These values provide a quantitative safety reference for the present AM stimulation regimen.

Notably, rhythmic TMAS-based electrical stimulation is inherently accompanied by rhythmic ultrasound stimulation. A substantial body of research has shown that ultrasound stimulation—through mechanisms such as acoustic radiation force—can effectively modulate neural activity [43,44]. Therefore, in AM-TMAS applications, the synergistic effects of combined acoustic and electrical stimulation should be taken into account.

In our electrical signal measurements, we employed saline with a conductivity of 4 S/m. Reported neural tissue conductivity values fall in the range of several to tens of S/m [45], and thus 4 S/m was selected to approximate the electrical properties of neural media while maintaining a stable and reproducible experimental environment. We also found that higher-conductivity saline solutions were more prone to bubble generation, which interferes with reliable detection. At this stage, our goal is to verify AM-TMAS feasibility in a homogeneous medium; subsequent studies will consider realistic skull and brain heterogeneity to evaluate how transcranial propagation and tissue inhomogeneity affect AM signal fidelity.

This study still has several limitations. First, the present magneto-acoustic electrical measurements were performed in saline with a conductivity of 4 S/m, which provides a stable and reproducible experimental environment but does not fully capture realistic skull and brain heterogeneity in transcranial applications. Second, the current work mainly provides engineering-level validation of waveform generation, inter-channel consistency, and magneto-acoustic electrical responses, rather than direct in vivo electrophysiological or behavioral evidence of rhythm-specific neuromodulation. Third, because rhythmic TMAS-based electrical stimulation is inherently accompanied by rhythmic ultrasound stimulation, the relative contributions of the acoustic and electrical components were not separately evaluated in the present study.

For future work, validation should be extended to more realistic transcranial conditions, and the smoothness of extremely low-frequency envelopes can be further improved. The current implementation uses a 64-point envelope LUT; for envelopes below 5 Hz, increasing the LUT point count may provide finer envelope sampling and smoother updates while maintaining reliable and repeatable waveform generation. In addition, subsequent animal experiments may include dose–response studies in small-animal models, combining local field potential (LFP) or multi-electrode recordings with behavioral assessments to clarify how different envelope frequencies (e.g., 8 Hz vs 40 Hz) differentially affect neural oscillations and behavior, ideally with comparisons against pure TUS or tACS modalities. Existing TMAS animal studies already provide preliminary evidence supporting such experiments.

## 5. Conclusions

We designed and implemented a 64-channel AM-TMAS driving system based on an FPGA with high-speed DACs, enabling synchronized phased-array excitation through a unified clock and a global UPDATE trigger. A programmable AM synthesis scheme (DDS + LUT with fixed-point envelope multiplication and center–multiply–restore mapping) supports carrier frequencies from 100 kHz to 2 MHz and envelope frequencies from 0.1 Hz to 100 kHz. Output tests confirm accurate and stable waveform generation (e.g., a 500 kHz setting measured at 499.998 kHz) with high envelope fidelity (NRMSE 1.0795% at 8 Hz and 1.9212% at 40 Hz for a 500 kHz carrier) and deterministic multi-channel alignment across the full array, with a worst-case inter-channel timing skew on the order of 5 ns (≈0.9° at 500 kHz). In physiological saline under a static magnetic field, the measured magneto-acoustic electrical response exhibits the intended 40 Hz rhythmic envelope (peak envelope correlation of 0.9919 after envelope extraction). Overall, the proposed platform provides a practical hardware basis for rhythm-targeted magneto-acoustic stimulation research; future work will further improve sub-Hz envelope smoothness and extend validation toward transcranial-relevant coupling and safety reporting (e.g., MI/TI) under specific transducer operating conditions.

## Data Availability

The original contributions presented in the study are included in the article, further inquiries can be directed to the corresponding author.
